# Exploring the Discursive Emphasis on Patients and Coaches Who Participated in Technology-Assisted Diabetes Self-management Education: Clinical Implementation Study of Health360x

**DOI:** 10.2196/23535

**Published:** 2022-03-18

**Authors:** Muhammed Y Idris, Ernest Alema-Mensah, Elizabeth Olorundare, Mohammad Mohammad, Michelle Brown, Elizabeth Ofili, Priscilla Pemu

**Affiliations:** 1 Department of Environmental Medicine and Public Health Icahn School of Medicine at Mount Sinai New York, NY United States; 2 Department of Community Health & Preventive Medicine, Morehouse School of Medicine Atlanta, GA United States; 3 Clinical Research Center Morehouse School of Medicine Atlanta, GA United States; 4 Morehouse Choice Accountable Care Organization and Education System, Inc, Morehouse School of Medicine Atlanta, GA United States; 5 Department of Medicine, Morehouse School of Medicine Atlanta, GA United States

**Keywords:** self-management, structural topic modeling, coaching, diabetes, minority populations, mobile phone

## Abstract

**Background:**

A critical unmet need for underserved patients with diabetes is regular access to sufficient support for diabetes self-management. Although advances in digital technologies have made way for eHealth applications that provide a scalable path for tailored interventions for self-management of chronic conditions, health and digital literacy has remained an obstacle to leveraging these technologies for effective diabetes self-management education. Studies have shown that the availability of coaches helps to maintain engagement in internet-based studies and improves self-efficacy for behavior change. However, little is known about the substances involved in these interactions.

**Objective:**

This study aims to compare the content of conversations between patient–coach pairs that achieved their self-management goals and those that did not. The context is a clinical implementation study of diabetes self-management behavior change using Health360x within the practices of the Morehouse Choice Accountable Care Organization in the Atlanta metro area. Health360x is a coach-assisted consumer health information technology designed to support self-management skills acquisition and behavior among underserved, high-risk patients with diabetes.

**Methods:**

We provide a novel analysis of the discursive emphasis on patients and coaches. We examined transcripts of visits using a structural topic model to estimate topic content and prevalence as a function of patient and coach characteristics. We compared topics between patient–coach pairs that achieved diabetes-related self-management goals and those who did not. We also estimated a regression in which utterances are the units, the dependent variable is the proportion of an utterance that is about a given topic, and the independent variables are speaker types and explored other themes.

**Results:**

Transcripts from 50 patients who were recruited and consented, starting in February 2015, were analyzed. A total of 44 topics were estimated for patient–coach pairs that achieved their intended health goals and 50 topics for those who did not. Analysis of the structural topic model results indicated that coaches in patient–coach pairs that were able to achieve self-management goals provided more contextual feedback and probed into patients’ experience with technology and trust in consumer information technologies. We also found that discussions around problem areas and stress, support (*β*_Coach_=.015; *P*<.001), initial visits (*β*_Coach_=.02; *P*<.001), problems with technology (*β*_Coach_=.01; *P*<.001), health eating goals (*β*_Coach_=.01; *P*=.04), diabetes knowledge (*β*_Coach_=.02; *P*<.001), managing blood sugar (*β*_Coach_=.03; *P<.*001), and using Health360x (*β*_Coach_=.003; *P*=.03) were dominated by coaches.

**Conclusions:**

Coach-facilitated, technology-based diabetes self-management education can help underserved patients with diabetes. Our use of topic modeling in this application sheds light on the actual dynamics in conversations between patients and coaches. Knowledge of the key elements for successful coach–patient interactions based on the analysis of transcripts could be applied to understanding everyday patient–provider encounters, given the recent paradigm shift around the use of telehealth.

## Introduction

e-Patients are a relatively new breed of informed health consumers comprising 61% of all adults in the United States using technology to *equip, enable, empower, and engage* with relevant health-related information [1]. Despite increasing access to wireless and mobile technology among minority and vulnerable populations, there is still a higher trend in the adoption of digital health technologies among disproportionately advantaged groups, such as young, White males [1-3]. It is known and well-accepted in the disparities research community that health and digital literacy are barriers to the increased use of eHealth technologies among minority and vulnerable populations. These obstacles include lack of perceived value, technologies creating more work, materials not in appropriate reading levels to make informed health decisions, lack of cultural relevance (ie, accessing sources in their respective language), and privacy and trust concerns [4]. These barriers are a function of individual characteristics, including socioeconomic status, education, and less access to change agents, among other factors that are associated with higher rates of attrition in internet-based studies [5-7]. Higher rates of attrition are even more noticeable among minority and underserved populations, who are also disproportionately challenged by intervention factors, including usability problems, complexity, and ease of discontinuance [4]. The net result is a lack of diffusion of innovations among vulnerable populations and a worsening of disparities in health outcomes [8], that is, the uneven adoption of digital health innovations by minority and other vulnerable populations because of health and digital literacy exacerbates existing disparities in health.

An area where there is an agreement in the literature is that health and digital literacy are obstacles to the use of eHealth technologies for implementing tailored health interventions that support self-management of chronic conditions, particularly diabetes [7]. A typical example for diabetes is the challenge with the delivery of appropriate and effective diabetes self-management education (DSME) to vulnerable populations. Multiple studies have found that diabetes DSME is associated with improved diabetes knowledge and self-care behavior [9], improved clinical outcomes such as lower hemoglobin A_1c_ [10], lower self-reported weight [11,12], improved quality of life [13], healthy coping [13,14], and lower costs [15]. Diabetes education is associated with increased use of primary and preventive services and lower use of acute, inpatient hospital services [16], and patients who participate in DSME are more likely to follow best practice treatment recommendations, particularly among the Medicare population. A critical issue and unmet need for underserved patients living with diabetes is the regular access to sufficient support for effective diabetes self-management. Better outcomes were reported for DSME interventions that were longer and included follow-up support, that were culturally appropriate and age appropriate, and that were tailored to the individual psychosocial needs and behavioral preferences [17,18].

Prior studies indicate that the availability of a trusted partner (coach) helps to improve user experience with health technology for DSME, including perceived relevance and ease of use [19], among vulnerable populations who face health and digital literacy barriers and higher rates of attrition. The support of a coach improves engagement with technology, and accountability improves adherence to and self-efficacy for behavior change [20]. In this patient–coach relationship, coaches are not intermediaries between patients and health information but serve as an apomediary, that is, a guide to relevant information and services for the patient [19]. Much has been theorized about what leads to successful patient–coach relationships; however, what is less understood is the content of patient–coach interactions.

In this paper, we aim to evaluate the discursive emphasis on patients and coaches engaged in DSME. To do so, we will leverage self-reported patient data and >100 hours of recorded transcripts from a clinical implementation study of Health360x, a coach-assisted consumer health information technology (CHIT) designed to support diabetes self-management skills acquisition and behavior for underserved, high-risk patients living with diabetes [19]. The platform is available as a web application and mobile app and includes functionality for improving health literacy and self-efficacy through access to a health coach, a social network of peers, a curriculum of DSME materials, and a health tracker that can record blood pressure, BMI, physical activity, and self-management goals.

In what follows, we provide some background on the study from which the data were generated. We then provide a novel analysis of the discursive emphasis on patients and coaches from transcripts of visits using a structural topic model (STM) to estimate topic content (ie, characteristic words in a topic) and prevalence (ie, proportion of an utterance that is related to a topic) as a function of patient and coach characteristics. We conclude with a discussion of our results, limitations, and directions for future work on the use of coach-assisted CHIT for DSME.

## Methods

### Transdisciplinary Collaborative Center Project

Longitudinal data and transcripts of patient–coach interactions were taken from a clinical implementation study of Health360x, a coach-assisted CHIT designed to assist with chronic illness care through behavior change [19,21]. It is important to note that as an implementation study, there was no intervention or control arm as the goal of the study is not to make generalizations to a broader population [22]. The research design of the study focused almost exclusively on external validity. As such, countless hours were spent working with clinical practices through focus groups and plan-do-study-act cycles to ensure clinical readiness. The goal of the transdisciplinary collaborative center was to study the implementation across several clinics that are part of the Morehouse Choice Accountable Care Organization (MCACO).

Health360x was developed at the Morehouse School of Medicine and evaluated within the MCACO. MCACO is a physician-led integrated delivery model participating in the Medicare Shared Savings Program offered by the Centers for Medicare and Medicaid Services Innovation Center [23]. The MCACO partner practices care for a disproportionate share of high-need, complex populations and endure extraordinary challenges in managing the use, with comparatively limited resources. The Health360x implementation at MCACO was nested within the MCACO’s Centralized Care Coordination model. This synergistic approach represents a *health system* where an African American majority patient population spends time and receives primary health care.

To determine health practice readiness for inclusion in the study, key individuals within the practice were identified for a focus group. For a clinic to participate, their front office manager, practice manager, lead physician, and (optionally) patients living with diabetes were required to participate in this focus group. The goal of this focus group was to delineate patient experiences and practice workflow for patients living with diabetes. Findings from focus group discussions were then used to identify and address issues around recruitment, signup, and visits through a plan-do-study-act cycle from select practices. In total, we recruited between 3 and 5 individuals across 5 sites for a total of 25 individuals for these focus groups.

Health coaches from within and outside of clinical practices were also recruited. The preferred characteristics included the following: (1) certified by Americans With Disabilities Act with the tool *Fundamentals of Diabetes Care*; (2) previous experience with living with diabetes either directly or through providing care for a family member or patient; (3) health care professionals with previous experience in educating patients living with diabetes; and (4) soft skills, including being considerate, responsible, dependable, and understanding within their community. A total of 4 health coaches were recruited by participating in health practices. This included 3 foreign-trained physicians, including 1 man in his late 30s whose second language was English, and 2 female physicians in their late 30s who were native English speakers. The final coach was a licensed practical nurse in her late 50s. All coaches identified as Black, with 50% (2/4) of coaches identifying as African and the others as African American.

Health coaches interacted with a slate of patients identified through the MCACO practices and established communication with the practice to facilitate patient self-management support. Approximately 20-30 patients across 4 practices were selected, obtained consent from, and trained on the use of Health360x to report outcomes of self-efficacy; overall health, including blood pressure, blood glucose, exercise, sleep, and quality of life; and satisfaction with care. A total of 200 patients were screened for being at high risk of living with diabetes. A total of 100 patients were excluded as they did not meet the eligibility criteria for the study or were part of a clinical site that was ultimately deemed *not* practice ready, leaving 100 patients to be allocated the intervention.

### Ethics Approval

This study was approved by the Morehouse School of Medicine Institutional Review Board (approval number 674).

### Inclusion Criteria

Included in the study were adults living with diabetes at high risk of complications and mortality, as defined by the presence of obesity or overweight status (BMI>25 kg/m^2^); tobacco use; history of depression; systolic blood pressure >140 mm Hg and diastolic blood pressure >90 mm Hg; hemoglobin A_1c_ >7%; recent hospitalization or emergency room visit for uncontrolled diabetes or hypoglycemia; history of renal disease defined as estimated glomerular filtration rate <60 mg/mmol; and history of heart attack, angina, claudication, or cerebrovascular disease.

### Training

Health coaches were required to complete 4 sessions of training. These sessions included training on diabetes knowledge, diabetes management and prevention, cultural competencies of the target population, working with low-literacy populations, patient recruitment and retention, confidentiality, and familiarity with data collection tools. Coaches were also required to complete web training and certification by the American Association of Diabetes Educators titled *Fundamentals of Diabetes Care*. Finally, the coach training sessions also included hands-on training on Health360x.

A total of 6 hours of training sessions were required to be completed by patients before the start of participation. The training was conducted at the Morehouse School of Medicine and covered the role of health coaches as navigators; the use of Health360x, glucose meter, blood pressure monitor, and pedometer; and process for downloading data. Patients with access to a computer or personal smartphone compatible with Health360x were instructed on how to access or download the app. Those patients who did not have access to a computer or personal smartphone that is compatible with the mobile app relied on a kiosk at the practice that was accessible when they visited their coach. The Health360x application access and training were completed through a combination of YouTube videos, and a handbook detailing steps for using the application was also provided to patients.

### Patient and Coach Visits

The health coach met with patients at the practice during scheduled visits. The purpose of each visit was to advance the patients’ self-efficacy for self-management of their diabetes. During the initial visit, patients were asked to select individual diabetes-related self-management health goals around being active, healthy coping and eating, monitoring, problem solving, reducing risky behaviors, and taking medication. At each subsequent visit, coaches ascertained the barriers the patient experienced through the use of motivational interviewing. Once these barriers were identified, incremental steps to address them, which the patient suggested or agreed with, were made. Techniques such as behavioral contracting were incorporated into the process. All interactions between patients and coaches were between 20 and 40 minutes. All meetings were digitally recorded and transcribed using a third-party transcription service. As a rule, initial schedules for interactions were every 2 weeks for the first 2 months and then monthly for 4 months.

### Statistical Analysis

#### Primary Outcome in Transdisciplinary Collaborative Center Study

The primary outcome of interest in the study was self-efficacy for behavior change, which is a predictor of intention and behavior. Self-efficacy was measured using a self-reported 10-point General Self-Efficacy Scale at each follow-up visit. A patient was determined to have achieved their self-efficacy goals if they self-reported a score ≥7.

#### Sample Size

Our sample size calculation was based on a systematic review and meta-analysis that included 85 high-quality published studies of internet-based behavioral health interventions with a total sample size of 43,236 patients [24]. On average, interventions with more extensive use of theory were associated with increases in effect size (*P*=.049). Interventions based on the theory of planned behavior tended to have substantial effects on behavior (Cohen *d*=0.36, 95% CI 0.15-0.56). Health360x was designed with particular attention to the incorporation of theory-driven behavioral change techniques such as an enriched information environment (current diabetes knowledge and curriculum), self-monitoring with color-coded feedback, goal setting, identifying barriers, and problem solving. Our a priori power analysis indicated that a minimum sample size of 97 was required to detect an anticipated effect size of 0.36, assuming a desired power and significance level of 0.8 and 0.05, respectively. Therefore, we recruited 100 patients for this study.

#### Topic Modeling

To explore the substance of conversations between patients and coaches, we used a topic model. This statistical machine learning method identifies common themes in a corpus of documents, or in our case transcripts, and is based on the intuition that particular words are associated with particular topics. Formally, each utterance in a conversation is represented as a vocabulary multiset with corresponding word counts (also referred to as a document-term matrix). Each word in this vocabulary has some associated probability that it belongs to a given topic. If we think of a conversation as representing a random mixture of topics that are in turn defined by a set of characteristic, high-probability words, we can infer latent topics.

**Figure 1:**
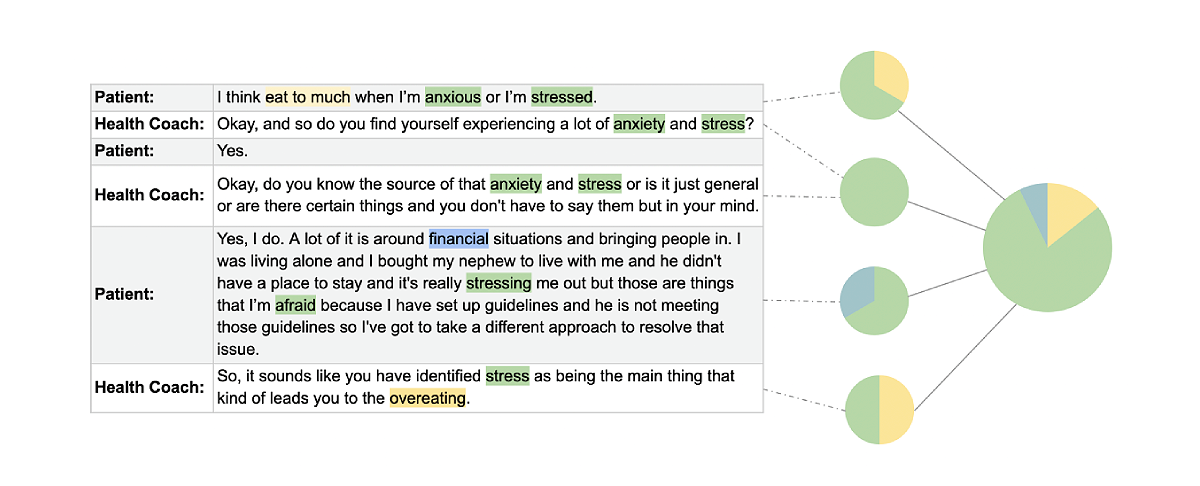
Example topic proportions and assignment.

Topic modeling has been shown to reliably uncover conversational topics from transcripts between patients and providers in clinical settings [25,26]. Figure 1 provides a visual example of how this works from a snippet of the conversation between a patient and a health coach. Characteristic words are highlighted in yellow, green, and blue and correspond to eating, anxiety, and finances, respectively. Taken together, we can see that this conversation is about anxiety and stress, leading to overeating. However, the snippet can be further broken down, and a topic model estimates the proportion of the conversation that corresponds to each of these topics based on the observed vocabulary.

We use an STM [27] for 2 main reasons: first, the STM extends traditional probabilistic models such as the Blei et al [28] latent Dirichlet allocation and the Blei and Lafferty [29] Correlated Topic model to incorporate document metadata into the analysis of latent topics. This was done by defining a generative process that produces *documents*, that is, utterances, with associated data, given document-topic and topic-word distributions, and using a variational optimization algorithm to estimate topic model parameters. Each conversation and utterance was made up of different proportions of the estimated topics. Incorporating speaker metadata into our topic modeling allowed us to explicitly model the association among patients, coaches, and topics of conversation, that is, the way the characteristics of patients and coaches were associated with the proportion of a specific topic in a given utterance. In our topic model, we included the speaker’s age, gender, and race and ethnicity, as well as that of the person they are speaking with and whether the speaker was a coach. We estimated a regression in which utterances were the units, the dependent variable was the proportion of an utterance that is about a given topic, and the independent variables were speaker types.

The second reason we used the STM relates to what is a common subjective decision made when using topic models: the number of topics to estimate. This decision was generally arbitrary. To avoid any human-injected bias in our analysis, we leveraged a data-driven approach to select the number of topics based on the identification of *anchor* words that exist in a document only if the document is about a specific topic. The Mimno and Lee [30] algorithm automatically selects distinctive and probable anchor words from which a number of topics can be reliably estimated. As the authors of the STM indicate in their documentation of their software in R (R Foundation for Statistical Computing), the primary advantage of this approach is that the number of topics to be estimated is automatically selected by the algorithm.

## Results

### Patient Sample Characteristics

A total of 100 high-risk patients with diabetes were recruited, and they consented to participate in this study from February 2015 over 5 years. Of the 100 patients, 18 (18%) patients were lost to follow-up before starting any coaching sessions, leaving 82 (82%) patients with at least 1 and as many as 11 follow-up visits. A total of 195 patient–coach follow-up visits were completed throughout the study, with an average of 2.35 (SD 1.98) follow-up visits per patient and 4.79 (SD 5.77) goals per patient. Of the 82 patients, 3 (4%) patients specified 1 diabetes-related self-management health goals, 18 (22%) patients specified 2 goals, 15 (18%) patients specified 3 goals, 8 (10%) patients specified 5 goals, 4 (5%) patients specified 7 goals, 1 (1%) patient specified 8 goals and another 15 goals, and 1 (1%) remaining patient specified 24 goals. Of the 82 patients who participated in the study, transcripts were analyzed for 51 (62%) patients. Digital recordings for the remaining 38% (31/82) of patients were of bad quality and, therefore, were not uploaded or corrupted during transcription. Most patients were Black and African American (45/51, 88%) and between the ages of 45 and 65 years (40/51, 78%). Table 1 provides a complete breakdown of patient demographic characteristics. Textbox 1 provides a sample of diabetes-related self-management goals.

**Table 1:** Patient demographic characteristics (N=51).

Characteristics		Values, n (%)
**Age range (years)**		
	18-24	1 (2)
	25-34	3 (6)
	35-44	2 (4)
	45-54	22 (43)
	55-65	18 (35)
	>65	3 (6)
	No response	2 (4)
**Gender**		
	Male	25 (49)
	Female	26 (51)
**Race and ethnic background**		
	Asian or Pacific Islander	1 (2)
	Black or African American	45 (88)
	Hispanic or Latino	1 (2)
	White	2 (4)
	Indian or Alaskan Native	1 (2)
	No response	1 (2)
**Insurance**		
	No insurance	17 (33)
	Medicare	12 (24)
	Medicaid	15 (29)
	Dual eligible	5 (10)
	No response	2 (4)

**Frequency of the number of health subgoals.**

**Health subgoals**

- Exercise longer
- Cope with diabetes
- Get support from my medical team
- Get support from my family and friends
- Get preventive help
- Stop smoking
- Check my feet
- Lose weight
- Get blood pressure under control
- Learn to have a safe pregnancy
- Follow my eating schedule better
- Eat better food
- Overeat less often
- Check my blood sugar more often
- Miss fewer blood sugar checks
- Do my blood sugar checks on time more often
- Prevent high blood sugars
- Treat high blood sugars
- Prevent low blood sugars
- Treat low blood sugars
- Manage diabetes when sick
- Miss fewer medications

### Structural Topic Model

#### Preprocessing

In total, our corpus included >17,000 talk turns, that is, an utterance or spoken statement in a conversation. To explore differences between topics of conversation between patient–coach pairs that achieved their health goals and those that did not, we split the corpus into successful patient–coach pairs (achieved) and patient–coach pairs that did not achieve the intended health goals (not achieved). This left us with 7196 talk turns in the achieved corpus and 9644 talk turns in the not achieved corpus. We then preprocessed each corpus using the textProcessor function in the STM R package to remove punctuation, stop words, numbers, and stemming. This produced a final document-term matrix with 6737 talk turns and a vocabulary of 1444 terms for the achieved group and 8663 talk turns and 1952 terms for the not achieved group. Patients represented more talk turns, that is, spoke more, in both the achieved and the not achieved groups, 60% (4318/7196) and 63% (6076/9644), respectively.

#### Topical Content

A total of 44 topics were estimated for patient–coach pairs that achieved their intended health goals and 50 topics for those who did not. In line with common practice, our approach to assigning labels to topics began by reviewing topic model outputs to identify potential topics based on the coherence of characteristic word forms. We then reviewed the high-scoring conversations for each topic to determine the topic labels. Where there was a clear topical theme in the first *N* talk turns, we validated the topic label. In general, a clear theme emerged within the first 30 talk turns. A review of high-scoring talk turns was completed collaboratively by 4 reviewers until a consensus on topic labels was reached. Of the 44 topics estimated for the achieved group, 13 (29%) coherent topics emerged, as presented in Textbox 2. Textbox 3 provides the results for the second STM of the not achieved group, which produced 10 coherent topics.

**Structural topic model 1 for patient–coach pairs that achieved health goals.**

**Topic 3: healthy eating**

- Eat
- Much
- Littl
- Meal
- Sometim
- Even
- Pretti
- Plan
- Ive
- Cook

**Topic 5: monitoring blood pressure and blood sugar**

- One
- Ten
- Twenti
- Hundr
- Fifteen
- Forti
- Twentieth
- Salad

**Topic 6: healthy coping**

- Diabet
- Feel
- Make
- Tell
- Confid
- Can
- Manag
- Enough
- Clear
- Prepar

**Topic 13: use of consumer health information technology**

- Use
- Password
- Ask
- Year
- Answer
- Comput
- Yesterday
- Long
- Past

**Topic 18: healthy snacking**

- Like
- Look
- Healthi
- Snack
- Always
- Challeng
- Per
- Made
- God
- Seem

**Topic 22: goal setting and habits**

- Want
- Mean
- Lot
- Youv
- Hous
- Line
- Note
- Never
- That’

**Topic 25: Health Insurance Portability and Accountability Act and diabetes knowledge (initial visit)**

- Health
- Inform
- Twelv
- Follow
- Account
- Reduc
- Accept
- Moham
- Higher
- Behavior

**Topic 26: sleep**

- Well
- Start
- Sleep
- Did not
- Went
- Bed
- Oclock
- Fill
- Sound
- Hurt

**Topic 30: monitoring for diabetics**

- Blood
- Sugar
- Check
- Morn
- Test
- High
- Prevent
- Weight
- Urin
- Cholesterol

**Topic 31: follow-up visit**

- Two
- Come
- Week
- Back
- Month
- That’s
- Appoint
- Thursday
- Within
- You will

**Topic 38: diabetes knowledge test**

- Glucos
- Exercis
- Insulin
- Low
- Blood
- Juic
- Lower
- Drink
- Fat
- Effect

**Topic 43: health coping and strategies (coaching)**

- Problem
- Stress
- Situat
- Serious
- Barrier
- Somewhat
- Live
- Import
- Way
- Facebook

**Topic 44: goal assessment and progress (coaching)**

- Said
- Anyth
- Continu
- Modifi
- Were not
- Alright
- Tomorrow
- Say
- That’
- Ive

**Structural topic model for patient–coach pairs that did not achieve health goals.**

**Topic 1: medication**

- Take
- Medic
- Medicin
- Side
- Took
- Wednesday
- Taken
- Have not
- Does not
- Gave

**Topic 2: problem areas, and stress and support**

- Feel
- Diabet
- Confid
- Umm
- Hot
- Track
- Level
- Control
- Comfort
- Live

**Topic 9: Health Insurance Portability and Accountability Act and diabetes knowledge**

- Help
- Health
- Care
- Inform
- Plan
- Idea
- Access
- Program
- Save
- Studi

**Topic 14: problems with technology**

- Use
- Way
- Password
- Pretti
- Email
- Step
- Happen
- Ahead
- Open
- Somewher

**Topic 15: healthy eating goals**

- Eat
- Hmm
- Goal
- Food
- Bettwe
- Set
- Schedul
- Term
- Active
- Healthi

**Topic 20: diabetes knowledge test**

- Blood
- Much
- Glucos
- Pressur
- Insulin
- Test
- Drink
- Enough
- Juic
- Check

**Topic 21: social determinants**

- Year
- Type
- Famili
- Ago
- Friend
- Person
- Meet
- Hes
- Son
- Brother

**Topic 22: sleep**

- Might
- Sleep
- Night
- Bed
- Monday
- Past
- Friday
- Somebodi
- Wake
- Troubl

**Topic 44: managing blood sugar (coaching)**

- Sugar
- Also
- High
- Blood
- Low
- Read
- Class
- Prevent
- Knowlegd
- Impact

**Topic 49: using Health360x**

- Can
- Put
- Now
- Enter
- Sheet
- Consent
- Link
- Pro
- Mix
- Anytim

#### Coaching and Contextual Feedback

We found quite a bit of overlap between topics of discussion between the 2 groups (eg, healthy eating, diabetes knowledge, and sleep). However, we found more coherent, that is, meaningful, topics that capture coaching in the achieved group versus in the not achieved group. Further investigation of high-scoring talk turns and the broader conversation that they were a part of provides many examples of coaches providing contextual feedback and incorporating this feedback into the patient’s strategy. Multimedia Appendix 1 provides 2 examples of such conversations. In the first example, which is taken from a high-scoring conversation on healthy coping and strategies, we see that after the coach first probes the source of anxiety and stress that is leading to overeating, acknowledges the patient’s feelings, and contextualizes how their anxiety and stress lead to overeating, only then does the coach start discussing a plan to reduce overeating by focusing on the identified stressors. The next sample conversation was around healthy eating. The coach begins the conversation by recentering the patient around the goal of getting their blood sugar down through exercise and healthier eating habits. The conversation quickly zeroes in a specific challenge in implementing this strategy: not eating on a regular schedule as the patient was not hungry at regular mealtimes. Only then does the coach provide contextualized feedback around what, when, and how much to eat.

#### User Experiences With CHIT

Another clear theme in our analysis of the topic model results was conversations around user experiences with CHIT. We found that CHIT use in the not achieved group focused almost exclusively on technical issues and how to use Health360x. Multimedia Appendix 2 provides 2 examples from the not achieved group. In the first example, the coach expresses frustration with technical issues that require troubleshooting Health360x during a demonstration with a patient. The second example is from a conversation in which the patient and coach work together to reset the password (a common theme throughout the study was remembering passwords) and then updating the patient’s health record on Health360x.

Conversely, the topic on the use of CHIT in the achieved group contained multiple examples of coaches probing user experiences with technology. Multimedia Appendix 3 provides 3 examples of coaches asking probing questions on CHIT use in the achieved group. These questions ranged from familiarity with technology and use of the internet to how trusting the patients are of consumer information technologies, including sharing their health and other information on those tools. What we found to be particularly interesting is that we did not find a topic in the achieved group that resembled issues with using Health360. This suggests that understanding a patient’s experiences with technology can lead to better coaching around the use of technology. This, in turn, can lead to better engagement with CHIT, self-management behavior changes, and health outcomes.

#### Who Dominates Topics of Conversation: Patients or Coaches?

In the achieved group, we found that healthy eating was much more likely to be discussed by patients. The average proportion of conversations about healthy eating across all patient and coach interactions, that is, E (*topic prevalence*)*,* was 0.026 or 2.6%. Being a coach was associated with an increase of 4.9% (*β*_Coach_=.049; *P*=.02) in the healthy eating topic proportion of a given utterance. The average proportion of conversations around follow-up visits and coaching in the form of goal assessment and progress across all visits was 3.4% and 0.8%, respectively. Being a coach was associated with an increase of 3% (*β*_Coach_=.03; *P*=.007) and 0.3% (*β*_Coach_=.003; *P*=.01) in the proportion of utterances related to follow-up visits and coaching in the form of goal assessment and progress. All other coherent topics in the achieved group were equally as likely to be discussed by either coaches or patients.

Conversations in the not achieved group were dominated by coaches. This included a 1.5% increase in the proportion of an utterance on problem areas, and stress and support (*β*_Coach_=.015; *P*<.001; E [topic prevalence]=0.012), a 2.4% increase related to initial visits (*β*_Coach_=.024; *P*<.001; E [topic prevalence]=0.012), a 1.3% increase around problems with technology (*β*_Coach_=.013; *P*<.001; E [topic prevalence]=0.012), a 0.8% increase in health eating goals (*β*_Coach_=.008; *P*=.04; E [topic prevalence]=0.03), a 1.7% increase in the proportion of diabetes knowledge (*β*_Coach_=.017; *P*<.001; E [topic prevalence]=0.02), a 3% increase in speaking about managing blood sugar (*β*_Coach_=.03; *P*<.001; E [topic prevalence]=0.02), and a 0.3% increase in speaking about using Health360x (*β*_Coach_=.003; *P*=.03; E [topic prevalence]=0.03). A total of 2 exceptions were the proportion of an utterance on medication (*β*_Coach_=−0.008; *P*=.009; E [topic prevalence]=0.02) and social determinants (*β*_Coach_=−0.02; *P*<.001; E [topic prevalence]=0.012), which were positively associated with patients.

**Figure 2:**
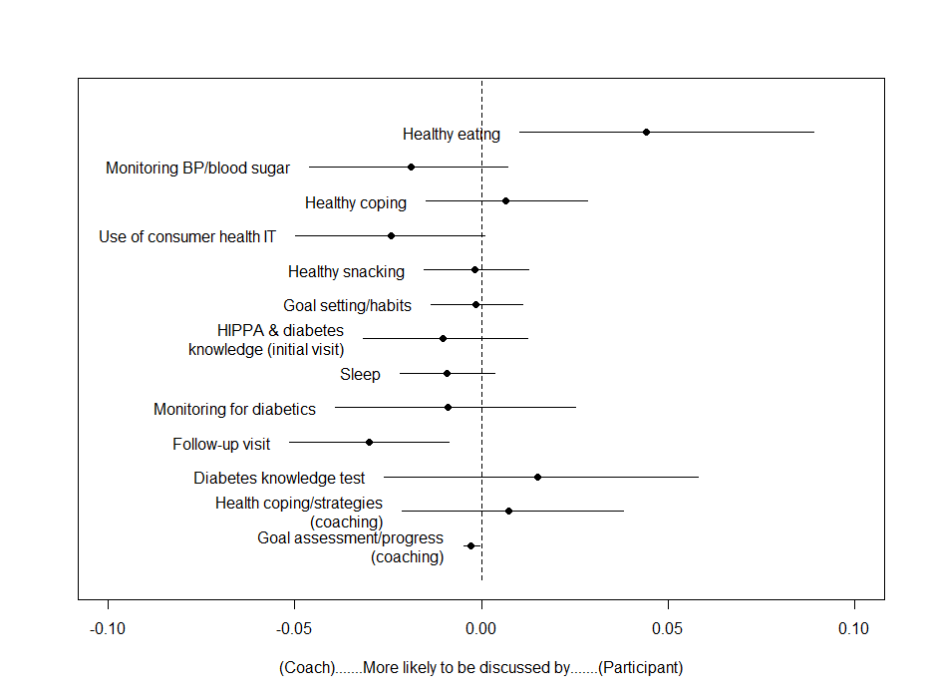
Mean difference in achieved topic proportions—patients versus coaches. BP: blood pressure; HIPAA: Health Insurance Portability and Accountability Act; IT: information technology.

**Figure 3:**
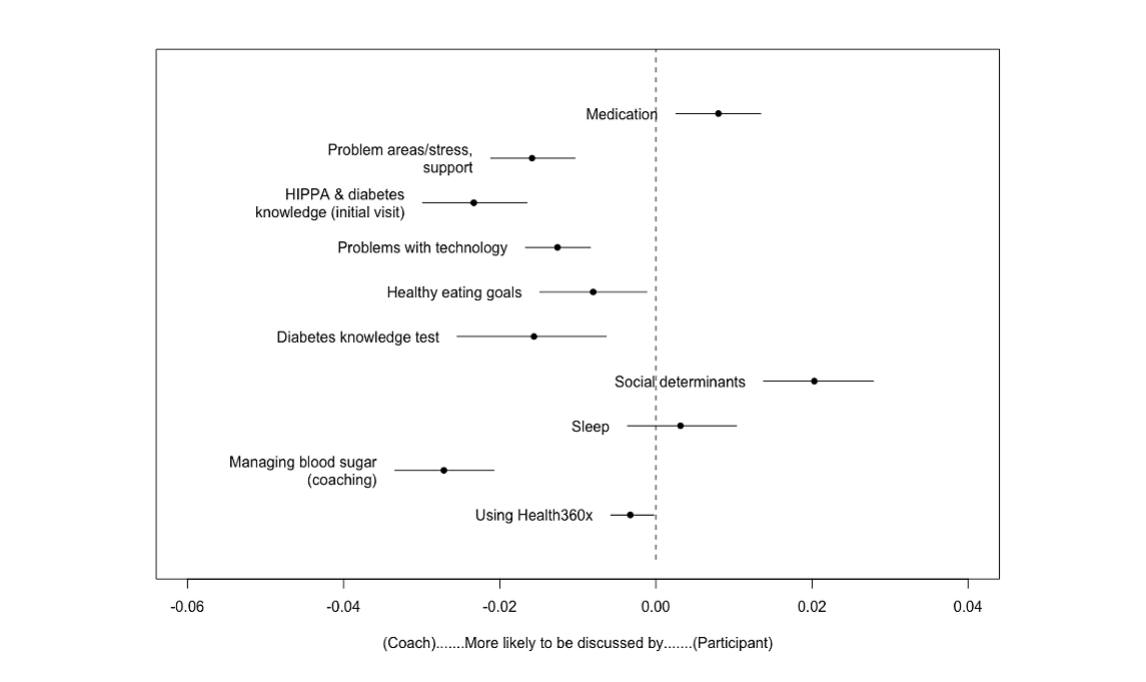
Mean difference in not achieved topic proportions—patients versus coaches. HIPAA: Health Insurance Portability and Accountability Act.

Figures 2 and 3 present the mean difference in topic proportions as a function of whether the speaker was a coach or a patient, that is, which topics were more likely to be discussed by coaches versus patients. Also included in the plots are CIs indicating a statistically significant difference between the 2 groups. Although we found quite a bit of overlap between topics of discussion between the 2 groups, plotting the mean difference in topic proportions across all coherent topics indicated that conversations in the achieved group were equally distributed between patients and coaches. However, topics of conversation between patients and coaches in the not achieved group did not include 0 in their 95% CIs, indicating that these topics were dominated, and therefore more likely to be driven, by coaches.

## Discussion

### Principal Findings

In this paper, we present the results of a novel analysis of the discursive emphasis on patients and coaches. The dialog is derived from an implementation of Health360x, a coach-assisted CHIT designed to support diabetes self-management skills acquisition and behavior for underserved, high-risk diabetic patients. Thematic analysis of conversations clustered using STM results of patient–coach interactions indicates that in patient–coach pairs that were able to achieve self-management goals, coaches provided more contextual feedback and probed into patients’ familiarity with technology and use and trust of consumer information technologies. We also found that topics of conversation between successful patient–coach pairs were equally distributed, whereas discussions with patients who were unable to achieve enhanced self-efficacy for diabetes-related self-management were dominated by coaches.

### Limitations

There are a few limitations of the analysis presented in this paper. First is the small corpus of transcribed conversations. As with all machine learning algorithms, the more training data, the better the models perform. Typically, topic models are used on corpora with tens of thousands of talk turns; however, we analyzed significantly less (approximately 17,000) talk turns. With that said, the methodological novelty of this paper is the incorporation of covariates into our estimation of topic models using the STM. Although we had a smaller than expected corpus, we found that we could reliably identify latent topics in comparatively smaller corpora by incorporating metadata into topic modeling. Transcription of the sessions was also outsourced and included errors, which may have further limited the overall information content of the transcripts.

A second potential limitation is that there are subjective decisions that go into creating a topic model that can influence topic outputs. The number of topics estimated as well as decisions around preprocessing (eg, removing stop words, stemming, and lemmatization) can influence topic model coherence and stability [31-33]. We addressed the subjectivity of arbitrarily selecting a number of topics by automating the number of topics to be estimated; however, another approach is to estimate and compare topic models with different topic sizes for stability across models. Finally, evaluations of preprocessing on topic model outputs are needed within the context of the STM used in this paper.

Another limitation relates to the lack of power in our analysis. The sample size calculation of 97 participants for this study was based on an effect size with a *P* value that is effectively .05. Moreover, our analysis of discursive analysis included transcripts from only half of the estimated sample size. To assess whether patient characteristics influence retention and transcript quality, we ran a logistic model regressing whether we had at least one session recording of high enough quality for analysis of patient characteristics. The results are presented in Multimedia Appendix 4. Patient characteristics, including gender, age, insurance status, and willingness to use the internet, were not significantly different between patients whose session recordings were retained and those whose session recordings were not.

A final limitation was related to the training of coaches, which was naturally limited. Coaches did not receive extensive training in cognitive behavioral therapy or motivational interviewing. Our training built the coaches’ existing skills and provided additional tools to help with an appreciative inquiry. In the future, we will use these learnings to provide more uniform training for health coaches.

### Comparison With Prior Work

A need exists to better understand how DSME might be delivered within primary care, the outcomes that can be achieved, and the training and system changes needed as a result [34]. Effective face-to-face multicomponent interventions require skilled primary care workers and activated patients coupled with tailored training and self-help systems, resources, and materials [35]. These interventions for diabetes self-management are complex, and their effectiveness is in large part determined by dynamics within the patient–provider relationship [36,37]. Where these relationships are 1-sided, that is, focus on what the patient *should* be doing, and do not account for a patient’s circumstance, strategies for encouraging diabetes-related self-management behaviors will be ineffective [38].

Patients stand the best chance of developing skills to actively participate and take responsibility in the management of their chronic condition when they receive personalized coaching. Polonsky and Fisher [39] provided a conceptual model of how the frequency of feedback, personal meaningfulness, clarity, guidance and support, and patient characteristics influence diabetes-related feedback and affect patient engagement and behavior change. The way each of these elements should be personalized for diabetes-related coaching to be effective is beyond the scope of this comparison with prior work. What is relevant is that our analysis of the discursive emphasis on patients and coaches provides a novel approach for quantifying the substance of these relationships. Examples of coaches providing contextual feedback, probing patient experiences with technology, and engaging in back-and-forth conversations are all indicators of tailored coaching. However, what remains less clear is how we can relate these conversations to clinical outcomes and how we can quantify the additional benefit that technology provides in addition to tailored coaching. These 2 questions are the focus of subsequent projects and manuscripts.

Our study also contributes to prior work by providing new and important insights into the usability and effectiveness of mobile health interventions targeting older African American patients living with diabetes. This underserved population has traditionally been considered not amenable to the use of technology to manage their health [4]. In addition, we present novel methods that can be used to understand (and predict) what is happening in patient–coach interactions. These methods enable the analysis of themes in text at scale using techniques that minimize the amount of human-injected bias into qualitative studies.

### Conclusions

Coach-facilitated, technology-based DSME can help address critical issues and unmet needs for underserved patients with diabetes around accessing sufficient support regularly. A variety of factors influence the efficacy of coaching; however, cultural expectations must be further explored, given the growing diversity among patients and the health care workforce prevalent today. Our use of structural topic modeling in this application is novel, and it creates an opportunity to introduce this technique into everyday patient–provider encounters, particularly in a post–COVID-19 world in which there has been a paradigm shift around the use of telehealth. The opportunity to create outputs that guide further physician action and patient action could drive better patient engagement and overall patient health outcomes.

## Appendix

Multimedia Appendix 1 Example of contextual feedback in health coaching around health coping and strategies.

Multimedia Appendix 2 Example of consumer health information technology problems and use in the not achieved subgroup.

Multimedia Appendix 3 Example of probing questions around consumer health information technology use in the achieved subgroup.

Multimedia Appendix 4 Logistic regression of at least one recording on patient characteristics.

## Abbreviations

CHIT: consumer health information technology
DSME: diabetes self-management education
MCACO: Morehouse Choice Accountable Care Organization
STM: structural topic model
