# Supplementary material for: Exploring the Discursive Emphasis on Patients and Coaches Who Participated in Technology-Assisted Diabetes Self-management Education: Clinical Implementation Study of Health360x
Source: J Med Internet Res. 2022 Mar 18;24(3):e23535. doi: 10.2196/23535 (PMC8976255; doi:10.2196/23535)
Supplement: Multimedia Appendix 1 [file jmir_v24i3e23535_app1.docx]

## Multimedia Appendix 1

Table S1. Example of Contextual Feedback in Health Coaching around Health Coping / Strategies

| Participant 044 | I think some of the things that make me overeat is anxiety and stress. |
| --- | --- |
| Health Coach | Okay, and so do you find yourself experiencing a lot of anxiety and stress? |
| Participant 044 | Yes. |
| Health Coach | Okay, do you know the source of that anxiety and stress or is it just general or are there certain things and you don't have to say them but in your mind. |
| Participant 044 | Yes, I do. A lot of my stress anxiety comes in around financial situations, people, I recently brought some people in. I was living alone and I bought my nephew to live with me and he didn't have a place to stay and it's really stressing me out but those are things that I can control because I have set up guidelines and he is not meeting those guidelines so I've got to take a different approach to resolve that issue. |
| Health Coach | So, it sounds like you have identified stress as being the main thing that kind of leads you to the overeating. |
| Participant 044 | And depressing because I didn't really think that I was getting depressed until I actually sat down and tried to do a daily review of things that was going on. Reflecting back to see why I am feeling this certain way. What is going on with me when I feel a certain way and a lot of it surrounds to stress and depression that sometimes comes over me because I feel like I'm not achieving some personal goals that I need to achieve. So, I try to do that. I try to meditate. I try to reflect. |
| Health Coach | Okay, and that’s really great because you have been able to on your own identify this is where this is coming from. This is where I'm not feeling good about this which is you know and again I know a lot of times in our country nobody wants to talk about depression and feeling some kind of weigh and they use all kinds of words like you're in a funk. You are not depressed that kind of a thing. But you know one step is identifying what exactly it is and realizing that it's something that comes along with human nature. As human beings we want to achieve certain things be it personal or be it other people’s expectations and when we feel that we are not achieving or measuring up to this it's natural to feel that depression or when there are things that are maybe outside of our control that we wish to be in control of but we can't so those kinds of things do bring on depression, stress, anxiety. |
| Participant 044 | Right. |
| Health Coach | And for some people, it leads to overeating, some people it leads to shopping all the time. It depends and so that's totally your experience and I think that in terms of trying to reduce overeating or overeat less often I think it's great that you've identified the stressors. |
| Participant 044 | Yes. |
| Health Coach | The next step would be as you have identified them try and see how you can make a plan to control some of them. To identify the ones probably that you know I can control the ones I may not be able to control and will I be okay with it. What am I ready to just let go without letting to cause me all this stress and so that's something that might help you with the overeating. |
| Participant 044 | Okay, so my additional goal would be a plan to minimize stress. |
| Health Coach | I think that would be great. That ties in very well with everything. |

| Table 6: Example of Contextual Feedback in Coaching around Healthy Eating | |
| --- | --- |
|  |  |
| Health Coach | Okay, so now we want to talk about your goal, do you know what your goal was? |
| Participant 077 | To get my sugar level down, to like a hundred. |
| Health Coach | Okay, so one of the ways you can do that is by of course taking your medication and another one is by... |
| Participant 077 | Exercising. |
| Health Coach | Excursing, and also eating healthy, so those are three goals we just made, but let’s start with healthy eating, now in the last the two weeks how would you rate you’re eating as far as eating schedule, meaning that you are eating frequently, maybe you have two snacks per a day, and you do that easting four a five vegetables a day per day. |
| Participant 077 | I love vegetable and fruits, |
| Health Coach | And you are eating on schedule you are not overeating... |
| Participant 077 | See that’s my big problem I never eat on schedule that what it is. |
| Health Coach | Okay. |
| Participant 077 | Like I got up at ten, I will eat three meals, I barely cane at two a day. |
| Health Coach | Why? |
| Participant 077 | My appetite is not used to it. so, I take a lot of medication I don't have an appetite to eat. |
| Health Coach | So, you think your medication might be holding your appetite, is acting as an appetite suppression or something, so you can start maybe by not, are your meals heavy or are they light? |
| Participant 077 | When I eat breakfast, it would be a light breakfast... |
| Health Coach | Like what? |
| Health Coach | A little bit of green... I eat a little potion of greens, a barley, a piece of meat or something like that. now my diner whatever I cook I eat I eat a full, I eat a meal and I am done.... that whole meal through the night, when I wake up in the morning, my stomach will growl like I ate nothing. |
| Health Coach | So, make a... like you say you eat a [Unclear] [00:22:20.13] maybe you can add in the greens so that you can maybe eat three meals a day maybe eat just the Barley maybe some toast, and see if that would you take you until it’s time for you... let’s if you eat it... [Unclear] [00:22:42.09] |
| Participant 077 | See I cannot [Unclear] [00:22:43.08] if I ate, so I eat no latter then09:30, and that will be my breakfast and then I get hungry again by three or four o'clock. |
| Health Coach | So that’s a pretty big spread at night. |
| Participant 077 | Yeah... |
| Health Coach | So you might want to try eating even though it’s not in the light of breakfast the what you are eating and then maybe a in three hours, three to four and hours later then you go an eat something else, instead of eating the grits the barley and the toast maybe you can just eat one of those eat like a boiled egg maybe. |
| Participant 077 | Maybe a piece of toast. |
| Health Coach | Maybe a piece of toast and see how that works, just change the so that you can get your frequency in there and then you say you wake up in the morning you, just starved. |
| Participant 077 | Yeah... |
| Health Coach | So how would rate yourself on a scale of one to ten then, one meaning that you are sort of track and ten meaning that you have done everything that you are supposed to do. |
| Participant 077 | No, I know I am ff track because I don't eat on a regular schedule time, I only take my medicine on a scale, so I know I am off track. |
| Health Coach | So yes, in two weeks when you come back, we will try. we hope that you have changed, sort of alter the way you are eating, and let’s do, so let’s give you a two, since you say you are off track. |
