# Supplementary material for: Exploring the Discursive Emphasis on Patients and Coaches Who Participated in Technology-Assisted Diabetes Self-management Education: Clinical Implementation Study of Health360x
Source: J Med Internet Res. 2022 Mar 18;24(3):e23535. doi: 10.2196/23535 (PMC8976255; doi:10.2196/23535)
Supplement: Multimedia Appendix 2 [file jmir_v24i3e23535_app2.docx]

## Multimedia Appendix 2

Table S1. Example of Consumer Health IT Problems/Use in Not Achieved

| Health Coach | There we go, alright. So, we're going to get you signed into your Health 360 and the good news is the pretty soon before the end of the month hopefully, fingers crossed, we have some things with the app so we should have them worked out so that you can install and be able to use your app. |
| --- | --- |
| Participant 060 | Okay. |
| Health Coach | So, that's somewhere that you can put. |
| Participant 060 | Right I can't give it up. |
| Health Coach | The app? Yes, that's the thing. There were some things that needed to troubleshoot because you'll have the app but to be able to log in might be a bit. |
| Participant 060 | Yes. |
| Health Coach | So, you click on the login. Let's see. That one. The one that says Health 360 login. Yes, so we're having some glitches in sink into that but they're working on it so hopefully, we should be able to get that all sorted out soon so that you can use it. So, you could go on there and put in your Email. I have the mouse though. And the last time my mouse wasn't working but I got a new one. So, let me put this up. It'll be easier that way. Put in the Email that you use and the password. |
|  |  |
|  |  |
| Health Coach | I'm going to put on the little recorder and I'll just have you logged in. Do you still remember your password? |
| Participant 045 | I'm not sure. There is only one or two. |
| Health Coach | Okay, so I'm going to click on the log in here and let you put in what you know and then your password but if you did forget the password it can send you the link to reset it. Okay, great so we are in awesome. Alright, I only had set up and put in some of your values but I think there was some that... I can do for the hour. Sometime when there has been a lot of a search it just starts to mess it up so I'm sorry I'll have you go ahead and put that in again. Okay, so we are back in that. So, what I was trying to do is to see if you had any new values that we could update with like your blood glucose over the last night. |
| Participant 045 | I haven't been to the doctor but as far as for mine, my last one was... What was my last one, it wasn't too high? it wasn't bad. it was like 121 because it wasn't high because whatever reason I thought it was but it wasn't. |
| Health Coach | Okay. |
| Participant 045 | I want to say it was like 121 or 131 something like that. |
| Health Coach | Okay, do you recall what day that was? |
| Participant 045 | It was about four days ago. |
| Health Coach | So, Saturday. So, let's see if we can update that. And we put Saturdays date it was the ninth and it was in the morning or in the evening. |
| Participant 045 | Afternoon. |
| Health Coach | About afternoon. About what time? |
| Participant 045 | I don't remember. I guess between two-three something like that. |
| Health Coach | Okay, so we will enter two for now. And you said it was about 120. |
| Participant 045 | 121 |
| Health Coach | Okay, 121. So, that goes into your record. We will come back and finish that when I do that. Okay, let's get that noted down. Look at that. It's not supposed to give an error message. Alight, so it will not take up too much time. What we are going to do today is we'll go over your sleep chart. |
