# Supplementary material for: Exploring the Discursive Emphasis on Patients and Coaches Who Participated in Technology-Assisted Diabetes Self-management Education: Clinical Implementation Study of Health360x
Source: J Med Internet Res. 2022 Mar 18;24(3):e23535. doi: 10.2196/23535 (PMC8976255; doi:10.2196/23535)
Supplement: Multimedia Appendix 3 [file jmir_v24i3e23535_app3.docx]

## Multimedia Appendix 3

Table S1. Example of Probing Questions around Consumer Health IT Use in Achieved Subgroup

| Health Coach | You got a computer? |
| --- | --- |
| Participant 048 | Yes. |
| Health Coach | Where is your computer located? |
| Participant 048 | Home. |
| Health Coach | You use a computer in the past? |
| Participant 048 | Yes, and this is. |
| Health Coach | And we already know how to that. So I am not going to answer that, and you know how to use the internet right? |
| Participant 048 | On my phone.. |
| Health Coach | The computer? |
| Participant 048 | I watch [Unclear] [00:11:28.13] |
| Health Coach | Uhuh.... Okay you ever look on it about your health? |
| Participant 048 | Yes. |
| Health Coach | Use Internet? |
| Participant 048 | Yeah. |
| Health Coach | You tell me you check your emails. |
| Participant 048 | Every time I have to. |
| Health Coach | Alight, I am comfortable... Putting my health information on the internet? |
| Participant 048 | Yes, |
| Health Coach | You agree? |
| Participant 048 | Yes. |
|  |  |
|  |  |
| Health Coach | have you used the internet sites to help you with your diabetes care? |
| Participant 028 | No. |
| Health Coach | Have you used any tool based on technology to help you with your diabetes? |
| Participant 028 | No. |
| Health Coach | I have used the internet for banking? |
| Participant 028 | No. |
| Health Coach | I have used the internet from email? |
| Participant 028 | No |
| Health Coach | Chartrooms? No? |
| Participant 028 | No. |
| Health Coach | Okay I feel comfortable putting my health information on the internet, strongly agree, agree, undecided or neutral, disagree, I strongly disagree? |
| Participant 028 | Undecided. |
|  |  |
|  |  |
| Health Coach | I feel I felt constant use that, you do not use that so... So, what have you used as far as technology in the past to what could have help? |
| Participant 030 | Nothing. |
| Participant 030 | What is this Chit thing you keep giving me the third degree about? I don't know what that is. It's giving me lots of question. |
| Health Coach | Have you ever used an application like this before like Health 350? |
| Participant 030 | No. |
