# Supplementary material for: Exploring the Discursive Emphasis on Patients and Coaches Who Participated in Technology-Assisted Diabetes Self-management Education: Clinical Implementation Study of Health360x
Source: J Med Internet Res. 2022 Mar 18;24(3):e23535. doi: 10.2196/23535 (PMC8976255; doi:10.2196/23535)
Supplement: Multimedia Appendix 4 [file jmir_v24i3e23535_app4.docx]

Table S1. Logistic Regression of at least 1 recording on patient characteristics

|  | **Binary Dependent Variable:**  *1 = Patient had at least 1 session with Health Coach* | |
| --- | --- | --- |
|  | **Beta (se)** | **P-value** |
| Gender (1 = Male) | 0.76 (0.49) | 0.127 |
| Age | -0.02 (0.02) | 0.343 |
| Insurance Status | -0.23 (0.22) | 0.290 |
| Willingness to Use the Internet | 0.02 (0.23) | 0.945 |
| N=84  AIC=121.2 | | |
